# Supplementary material for: MiR-486-5p Targets CD133+ Lung Cancer Stem Cells through the p85/AKT Pathway
Source: Pharmaceuticals (Basel). 2022 Feb 28;15(3):297. doi: 10.3390/ph15030297 (PMC8951736; doi:10.3390/ph15030297)
Supplement: Supplementary file 1 [file pharmaceuticals-15-00297-s001.zip › Supplementary Legend.pdf]

Supplementary Figure Legend

Suppl. Figure S1: Effects of transfection in lung cancer cell lines. MiR-486-5p levels in lung cancer cell lines transiently **(A)** and stably **(B)** transfected with miRNA mimimc (mim-486). miR-486-5p relative expression vas measured by the –ΔΔCt method using RNU48 as housekeeping RNA and cells transfected with the control vector (ctrl-v) as calibrator. Experiements were run in triplicates. **(C)** Effects of the transfection of the ctrl-v on the percentage of CD133+ cells evaluated by flow-cytometry. Five replicates were performed for each experiment.

Suppl. Figure S2: Transient transfection of hsa-miR-486-5p miRNA mimic (mim-486) and the control vector in lung cancer cell lines. **(A)** Transfected cells were plated in standard medium and counted with trypan blue at 72 and 120 hours. Apoptosis was measured by flow cytometry as annexin Vpos/7AADneg cells 48h after mimic transfection and represented as a fold increase compared to cell transfected with the control vector. Graphs show percentage of different Cell cycle phases after mim-486 transfection. Cell **(B)** migration and **(C)** invasion capability was assessed by transwell assay at 24h. All data are expressed as mean ± standard error of the mean (SEM) of 5 biological replicates. Significant p-values <0.05 are indicated by an asterisk. **(D)** Xenograft models of the 4 transfected lung cancer cell lines: 500,000 cells were inoculated into the flanks of SCID mice. Data are expressed as mean ± standard error of the mean (SEM).

Suppl. Figure S3 Endogenous levels of miR-486-5p, p85 and CD133+ cells in lung cancer cell lines. Graphs shows relative expression of miR-486, p85 and frequency of CD133+ cells in H460, LT73, A549 and H1299 (n=3 for each cell lines). Data are expressed as mean ± standard error of the mean (SEM).

Suppl. Figure S4: Luciferase assay to evaluate the direct regulation of CD133 mRNA by miR-586-5p. Reporter systems were transfected in HEK293 with CD133 3'UTR containing vector in combination with the control vector (ctrl-v) and miR-486-5p mimic (mim-486). On the left the alignment of the miRNA seed sequence with the CD133 3'UTR predicted binding site. On the right Bar graphs showing average luciferase activity. Data are expressed as mean ± standard error of the mean (SEM).

Suppl. Figure S5: Evaluation of miR-486-5p distribution in mice organs. **(A)** Schematic representation of CCL-486 schedule of administration. **(B)** Quantification of miR-486-5p positive cells percentage in CCL-486 treated PDX111, at the end of the experiment. Av. = mean ± standard error of the mean (SEM) of all ten tumors. **(C)** DigitalPCR analysis of miR-486 levels in tumor, liver, lung, and spleen of untreated, control vector and CCL-486 treated PDX111. Data are expressed as mean ± standard error of the mean (SEM). **(D)** Ratio between main organs and mouse weight in non-treated (N.T), control and CCL-486 treated PDX111. Data are expressed as mean ± standard error of the mean (SEM).

Supplementary Table S1: miR-486-5p predicted target by miRWalk considering already validated targets included in miRTarBase and predicted also by TargetScan and miRDB databases.

Gene symbol	Binding probability	Energy	Accessibility	Number of pairings	Binding region length	Longest consecutive pairings	position	miRTarBase ID
**PIK3R1**	1	-23	0.011788	14	17	11	3UTR	MIRT735338
**PIK3R1**	1	-21.8	0.000674	15	19	7	CDS	MIRT735338
**CADM1**	1	-21.4	3.18E-06	13	14	13	3UTR	MIRT438847
**FBN1**	1	-26.6	0.000833	16	19	8	CDS	MIRT731367
**HAT1**	1	-20.5	7.35E-06	12	14	8	3UTR	MIRT735253
**SMAD2**	0.969231	-25.7	5.23E-05	18	23	7	3UTR	MIRT732557
**ARHGAP5**	0.923077	-20.7	0.009394	19	24	12	3UTR	MIRT053295
**CADM1**	0.923077	-24.1	0.000325	20	30	8	3UTR	MIRT438847
**CADM1**	0.923077	-21.4	3.18E-06	13	14	13	3UTR	MIRT438847
**CDK4**	0.923077	-22.6	2.22E-05	14	19	8	CDS	MIRT731583
**SMAD2**	0.923077	-28.9	0.000217	18	20	16	3UTR	MIRT732557
**FOXO1**	0.923077	-23	1.58E-05	16	22	9	CDS	MIRT734356
**HAT1**	0.923077	-20.5	7.35E-06	12	15	8	CDS	MIRT735253
**HAT1**	0.923077	-22.5	0.00091	21	31	8	5UTR	MIRT735253
**PIK3R1**	0.923077	-23	0.011788	14	17	11	3UTR	MIRT735338
**PIK3R1**	0.923077	-22	0.000195	15	19	8	3UTR	MIRT735338
**ARHGAP5**	0.846154	-21.3	5.77E-06	13	18	9	CDS	MIRT053295
**DOCK3**	0.846154	-25.8	3.66E-05	18	25	6	CDS	MIRT438814
**CADM1**	0.846154	-21.6	9.01E-06	19	28	6	3UTR	MIRT438847
**FBN1**	0.846154	-31.6	0.002803	19	26	10	CDS	MIRT731367
**SMAD2**	0.846154	-22.6	0.000862	20	41	8	3UTR	MIRT732557
**SMAD2**	0.846154	-22.1	0.001452	17	30	6	5UTR	MIRT732557
**SMAD2**	0.846154	-29.9	1.58E-06	16	20	10	3UTR	MIRT732557
**PTEN**	0.846154	-23.7	1.10E-07	21	49	13	5UTR	MIRT734357
**PIK3R1**	0.846154	-22	0.000195	15	19	8	3UTR	MIRT735338
**DOCK3**	0.807692	-22.3	0.008488	19	30	10	CDS	MIRT438814

Supplementary Table S2: Extreme Limiting Dilution Assay

Mim-486 transfected A549 cells

N. injected Cells	N. Injections	N. Grown tumors	Group
10000	12	10	ctrl
10000	12	6	486
100000	12	12	ctrl
100000	12	6	486
500000	12	12	ctrl
500000	12	10	486

Confidence intervals for 1/(stem cell frequency)			
**Group**	**Lower**	**Estimate**	**Upper**
**486**	**265554**	**148226**	**82736**
**ctrl**	**11308**	**5582**	**2755**

Overall test for differences in stem cell frequencies between any of the groups **P.value: 6.44e-12**
